# Brazilian Red Propolis Attenuates Inflammatory Signaling Cascade in LPS-Activated Macrophages

**DOI:** 10.1371/journal.pone.0144954

**Published:** 2015-12-14

**Authors:** Bruno Bueno-Silva, Dione Kawamoto, Ellen S. Ando-Suguimoto, Severino M. Alencar, Pedro L. Rosalen, Marcia P. A. Mayer

**Affiliations:** 1 Department of Microbiology, Institute of Biomedical Sciences, University of São Paulo, São Paulo, SP, Brazil; 2 College of Agriculture “Luiz de Queiroz” (ESALQ/USP), Piracicaba, SP, Brazil; 3 Piracicaba Dental School, University of Campinas–UNICAMP, Department of Physiologic Sciences, Piracicaba, SP, Brazil; Universitatsklinikum Freiburg, GERMANY

## Abstract

Although previous studies suggested an anti-inflammatory property of Brazilian red propolis (BRP), the mechanisms involved in the anti-inflammatory effects of BRP and its activity on macrophages were still not elucidated. This study aimed to evaluate whether BRP attenuates the inflammatory effect of LPS on macrophages and to investigate its underlying mechanisms. BRP was added to RAW 264.7 murine macrophages after activation with LPS. NO production, cell viability, cytokines profile were evaluated. Activation of inflammatory signaling pathways and macrophage polarization were determined by RT-qPCR and Western blot. BRP at 50 μg/ml inhibited NO production by 78% without affecting cell viability. *Cd80* and *Cd86* were upregulated whereas *mrc1* was down regulated by BRP indicating macrophage polarization at M1. BRP attenuated the production of pro-inflammatory mediators IL-12, GM-CSF, IFN-Ɣ, IL-1β in cell supernatants although levels of TNF- α and IL-6 were slightly increased after BRP treatment. Levels of IL-4, IL-10 and TGF-β were also reduced by BRP. BRP significantly reduced the up-regulation promoted by LPS of transcription of genes in inflammatory signaling *(Pdk1*, *Pak1*, *Nfkb1*, *Mtcp1*, *Gsk3b*, *Fos* and *Elk1*) and of *Il1β* and *Il1f9* (fold-change rate > 5), which were further confirmed by the inhibition of NF-κB and MAPK signaling pathways. Furthermore, the upstream adaptor MyD88 adaptor-like (Mal), also known as TIRAP, involved in TLR2 and TLR4 signaling, was down- regulated in BRP treated LPS-activated macrophages. Given that BRP inhibited multiple signaling pathways in macrophages involved in the inflammatory process activated by LPS, our data indicated that BRP is a noteworthy food-source for the discovery of new bioactive compounds and a potential candidate to attenuate exhacerbated inflammatory diseases.

## Introduction

Inflammation provides protection against pathogens, but also modulates repair and healing after cellular damage. In most human diseases, including auto inflammatory and autoimmune diseases, the fine balance between the insult and the host response is disrupted due to genetic and environmental factors, leading to inflammatory damage[1]. Inflammation may be controlled by non-steroidal anti-inflammatory drugs, but other treatment strategies include the administration of inhibitors of pro-inflammatory cytokines, such as anti- tumor necrosis factor alpha (TNF-α) [2], anti-interleukin (IL)-6 [3], and anti-IL-1 [1].

Macrophages exhibit multiple functions during the immune response [4]. In the context of inflammation, circulating monocytes are recruited and differentiate into macrophages [5]. Macrophages can be activated by a wide range of substances, including cytokines derived from T and natural killer (NK) cells and direct recognition by binding to microbial components such as the lipopolysaccharide (LPS) from the Gram negative bacteria cell wall. These highly plastic cells differentiate with substantial shifts in gene expression depending on specific stimuli, giving rise to at least two phenotypes with specialized functions[6].

The M1 phenotype is involved in phagocytosis, secretion of inflammatory cytokines and reactive compounds such as nitric oxide (NO)[7], and exhibits the surface markers CD 80 and CD86. M2 phenotype participates in tissue repair and regeneration [5], can produce regulatory cytokines such as IL-10, exhibits the CD206 surface receptor and produces arginase-1 [8].

Despite the protective role of inflammation in eliminating pathogens and promoting tissue regeneration, the exacerbated inflammatory process is involved in several diseases in humans, including cardiovascular diseases, diabetes, arthritis, inflammatory bowel disease and periodontitis, to mention only a few. Therefore, the search for new drugs or even functional foods that reduce the recruitment of neutrophils and macrophages in different models of inflammation, or alter the differentiation process of monocyte-derived macrophages, leading to different phenotypes, is intense in the literature[9, 10].

Natural products have been investigated as an alternative source of drugs which modulate the inflammatory process [11]. Propolis, a non-toxic resinous substance collected from various parts of plants as sprouts, floral buttons and resinous exudates by Africanized bees *Apis mellifera* [12] has been used extensively as additives in food and beverages due to its beneficial properties to human health and activity on diseases prevention.

Brazilian propolis has attracted scientific interest due to its several biological, pharmaceutical and nutraceutical properties such as antimicrobial, antibiofilm, anticaries [13, 14], antioxidant [15], anticancer[16]and anti-inflammatory [17, 18]. Propolis is formed by multiple components, in a wide chemical diversity and different types are characterized by distinct components [12, 19, 20]. Data on the anti-inflammatory effects of Brazilian propolis are abundant [21–25], however, there are few studies on the anti-inflammatory properties of the Brazilian red type [14, 17].

Our research group has previously determined the chemical composition of BRP [12, 14]. Lately, red propolis was shown to inhibit NO production and neutrophil migration into the peritoneal cavity of mice [17]. Despite the anti-inflammatory potential of BRP, little is known on the mechanisms involved in the regulation of inflammation induced by propolis. Therefore, we tested the hypothesis that BRP attenuates the macrophage response to bacterial lipopolysaccharide (LPS). LPS activated macrophages were submitted to BRP and their polarization determined by the secretion of NO and cytokines (IL-12p40, GM-CSF, IFN-γ, IL-1β, IL-10, TGF-β, TNF-α and IL-6) and transcription of 360 genes involved in the inflammatory process and surface markers. Furthermore, the activation of pathways involved in macrophages response to LPS and the expression of TIRAP, an upstream adaptor molecule involved in TLR4 signaling, were also determined.

## Materials and Methods

### BRP solution preparation

Red propolis was collected by scraping the insides of the boxes of *Apis mellifera* bees in the seaside region of Maceio, Alagoas, Brazil. The propolis was collected in a private land, whose owner gave permission to conduct the study. The crude extract was obtained by mixing 25g of propolis with 200ml of 80% ethanol (v/v). Then, crude extract was filtered using qualitative filter paper 80g, the solvent was evaporated and BRP was diluted in DMSO (1:500) at concentrations ranging from 40 to 100 μg/mL.

### Gas chromatography coupled to mass spectrometry (GC-MS)

The GC-MS analyzes were conducted on a Shimadzu gas chromatograph model GC 2010 coupled with mass spectrometry Shimadzu Model QP 2010 Plus equipped with a capillary column (RTX5MS 30m x 0.25mm x 0.25 μm). The initial column temperature was 80°C for 1 minute; reached 250°C by the rate of 20°C/min and kept at this temperature for 1 minute, from 250 to 300°C with rate of 6°C/min for 5 minutes; 300 to 310°C with rate of 15°C/min for 5 minutes; 310 to 320°C with rate of 20°C/min for 10 minutes, completing 40 minutes of analysis. Helium was used as carrier gas. The injector temperature was 280°C and the injection volume was 0.2 μL in splitless mode. The interface temperature was maintained at 280°C. The mass detector operated in mode scanning m/z from 40 to 800. The integration was done in software solution LabSolutions-GCMS and the identification of compounds was performed by comparison with the data of the Wiley mass spectrum library 8TM and authentic patterns injected under the same conditions of the samples[12].

### Growing of eukaryotic cell

RAW 264.7 cells have been established from murine tumors (leukemia) induced by the Abelson leukemia virus (Raschke et al., 1978). RAW 264.7 cells were cultured and maintained in DMEM medium (Cultilab, Campinas, Brazil) containing 10% fetal bovine serum and 1% antibiotic solution: 1,000U/mL penicillin G (ICN Biomedicals, Irvine, CA, USA) and 100U/ml streptomycin sulfate (Calbiochem, Darmstadt, Germany).

### LPS activation of macrophages in the presence of BRP

Cells (1x10^5^ cells/well) were activated with 10μL of lipopolysaccharide (LPS) from *E*. *coli* serotype O111:B4 (Sigma, St. Louis, MI, USA) at 500 ng/ml. At the same time, aliquots of BRP (40–100 μg/ml) were added to each well and the plates were incubated for 48 hours at 37°C in 5% CO_2_ with LPS and BRP or controls. Cells added with the vehicle (DMSO) with and without LPS and/or BRP were used as controls [26].

### Determination of the effect of BRP on NO production and cell viability

The production of NO was determined by measuring nitrite in cell culture supernatants. Cells supernatants were incubated with an equal volume of Griess reagent (Sigma, St. Louis, MI, USA), and the absorbance was determined at 540 nm. Results were expressed as mM of NO_2_.

Cell viability was determined by 3-(4,5-dimethylthiazol-2-yl)-2,5-diphenyltetrazolium bromide (MTT) (Sigma-Aldrich, St. Louis, MI, USA) assay.

### Cytokines quantification

Cytokines profile was determined in the supernatant of LPS activated macrophages submitted to 50μg BRP/ml since this condition led to the greatest reduction in NO levels without loss in cell viability. Data were compared with control LPS treated cells. Controls cells not treated with LPS, with and without BRP, were also used. Levels of IL-12, GM-CSF, IFN-γ, IL-1β, IL-10, TGF-β, TNF-α and IL-6 were determined by enzyme-linked immunosorbent assay (ELISA) using commercial kits (Becton-Dickinson, San Diego, CA, USA). Absorbance was determined at 450 nm and data expressed in ρg/ml.

### Gene expression

Gene expression was determined by reverse transcription followed by real time PCR. Total RNA was extracted from LPS activated RAW 264.7 macrophages submitted to 50μg BRP/ml and control LPS treated cells, in three independent experiments, using RNA extraction kit (Qiagen, Hilden, Germany). First strand synthesis was obtained with 1 μg of RNA using RT^2^ First Strand Kit (Qiagen). PCR was performed using arrays for mouse common cytokines (PAMM-021CZ), mouse Signal Transduction Pathway (PAMM 014CZ), mouse phosphoinositide 3-kinase- Protein kinase B (PI3K-AKT) Signaling Pathway (PAMM-058CZ) and nitric oxide signaling pathway (PAMM-062CZ) (Qiagen), totalizing 360 genes. Changes in gene expression of the target genes were measured relative to the mean cycle threshold (CT) values of five different calibrator genes (*gusb*, *hprt*, *hsp90ab1*, *gapdh* and *actb*) using the ΔΔCT method. Macrophages polarization at M1 or M2 was determined by measuring mRNA levels of *arg1*, *mrc1*, *cd80* and *cd86*, relative to levels of *gapdH* transcripts [26].

### Proteins detection by Western Blot

The amounts of phosphorylated proteins indicative of different pathways activation and of Tirap, an adapter of TLR 4, were determined in LPS activated RAW 264.7 macrophages submitted to 50μg BRP/ml and control LPS activated cells by Western Blot.

Cell lysates were prepared by re-suspending RAW 264.7 macrophages in SDS-PAGE loading dye (BioRad, Hercules, CA, USA) and boiling for 10 minutes. Protein concentrations were determined by the Bradford method and 30 μg of protein were loaded on 12% Bis-acrylamide -Tris gel. After electrophoresis, the proteins were transferred to a nitrocellulose membrane (Life Technologies). The membranes were blocked with 5% skim milk, and incubated with primary antibodies to the pNF-κB p65 (Ser536) (93H1), pC-Fos (Ser 32) (5348), p-p42/44 (phospho MAPK–p42/44 (Thr180 / Tyr182)-4631S) (Cell Signaling, Danvers, MA, USA), and Tirap (Invivogen 48–2300, San Diego, CA, USA) at 1: 1,000 dilution. Anti-GAPDH (2118) (Sigma-Aldrich, St. Louis, MI, USA) was used as the control antibody. After incubation with the secondary antibody at 1: 2,000 dilution (anti-rabbit IgG, Sigma-Aldrich), the detection was performed using "Prime Amersham ECL Western Blotting Detection" reagent (GE Healthcare, Uppsala, Sweden). The autoradiograms were photographed and bands intensity compared visually.

### Statistical Analysis

Differences in cell viability, NO and cytokines levels among the groups were determined using one-way ANOVA followed by Tukey, with the aid of Biostat Software. Student’s t-test was used to assess differences in gene transcription profiles between control and experimental groups using mean CT values. Differences of ≥ 5-fold change in gene expression were considered significant when p < 0.05, using SABiosciences Technical Core website (SABiosciences/Qiagen Corp., Frederick, MD, USA).

## Results

### Chemical analysis

**Fig 1:**
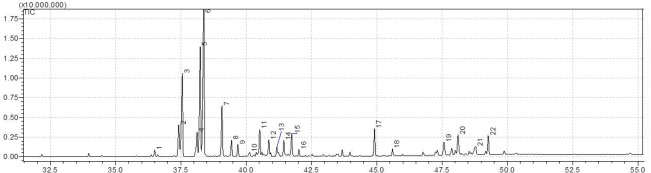
Chemical profile of BRP obtained by GC-MS. 1 4,4'bis[(trimethylsilyl)ethynyl]-2,2'-bithiophene-5,5' dicarbaldehyde; 2 silane, trimethyl[5-methyl-2-(1-methylethyl)phenoxy]; 3 medicarpin; 4 benzenepropanoic acid, 3,4-bis[(trimethylsilyl)oxy]-, trimethylsilyl ester; 5 neovestitol; 6 vestitol; 7 4,4'-bis[(trimethylsilyl)ethynyl]-2,2'-bithiophene-5,5'-dicarbaldehyde; 8 hydrocinnamic acid, p-(trimethylsiloxy)-, trimethylsilyl ester; 9 3,4-dihydroxy-9-methoxypterocarpan; 10 3,8-dihydroxy-9-methoxypterocarpan (3-hydroxy-8,9-dimethoxypterocarpan); 11 1,3,5-cycloheptatrien, 7-methyl-7-phenyl-2,4-bis(trimethylsilyl); 12 formononetin; 13 silane, 9h-fluoren-9-ylidenebis[trimethyl; 14 benzeneacetic acid, 2,4,5-tris[(trimethylsilyl)oxy]-, trimethylsilyl ester; 15 isoliquiritigenin; 16 2-propenoic acid, 3-(3,4,5-trimethoxyphenyl)-, methyl ester; 17 benzeneacetic acid, 4-[(trimethylsilyl)oxy]-, trimethylsilyl ester; 18 propanedioic acid, bis[(trimethylsilyl)oxy]-, bis(trimethylsilyl) ester; 19 Silane, trimethyl[[(3.beta.)-olean-12-en-3-yl]oxy]- $ $ 3-[(trimethylsilyl)oxy]olean-12-ene; 20 not identified; 21 not identified; 22 Lup-20(29)-en-3-yl acetate.

The chemical analysis by CG-MS revealed 22 distinct compounds in chemical composition of BRP. Most of these compounds are isoflavonoids and flavonoids, a group of isoflavones with recognized therapeutic properties. The most abundant chemical compounds are vestitol and neovestitol, both isoflavonoids (Fig 1).

### NO quantification and cells viability

**Fig 2:**
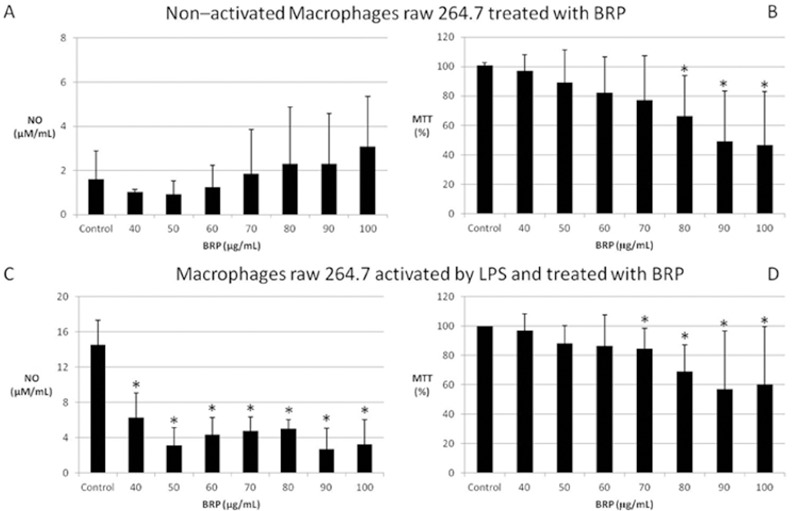
Effect of BRP treatment for 48 h on NO production (in A and C) and cell viability (in B and D) of RAW 264.7 non-activated cells (A and B) and activated with 500 ng/ml LPS (C and D). Results are expressed as means followed by standard deviation of three independent experiments performed in triplicate. (*) Indicates statistically significant difference compared to control (DMSO) group by Analysis of variance (One-way ANOVA, p <0.05).

Cell viability was not affect by BRP, except for the higher tested concentrations, as shown in Fig 2. However, NO production was reduced in LPS (500 ng/ml) treated cells even at the lower tested BRP concentration (Fig 2).

### Cytokines in cell supernatant

**Fig 3:**
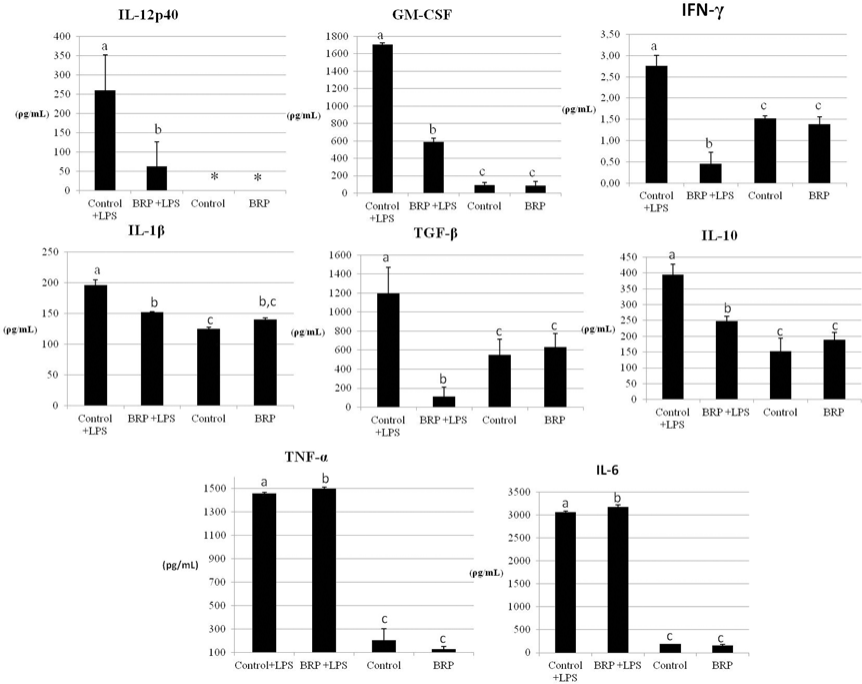
IL-12, GM-CSF, IFN-γ, IL-1β, IL-10, TGF-β, TNF- α and IL-6 levels (pg/ml) in the supernatant of LPS (500 ng/mL) activated RAW 264.7 cells treated with BRP (50 μg/mL in DMSO) for 48 hours. **Control + LPS: cells treated only with LPS and DMSO. Control: cells treated with DMSO. BRP: cells treated only with BRP.** (*) Indicates cytokine levels below the detection limit. Same letters mean no statistical difference while different letters mean statistically significant difference between the two bars by analysis of variance (One-Way ANOVA, p <0.05).

The lowest BRP concentration (50 μg/mL) which led to the highest NO reduction (78%) without loss in cell viability was used to evaluate the cytokines profile in LPS treated macrophages (Fig 2). LPS activation resulted in the production of all the studied cytokines. The BRP treatment on LPS activated macrophages inhibited the production of IL-12, GM-CSF, IFN-γ, IL-1β, IL-10 and TGF-β. On the other hand, BRP treatment led to a slight but significant increase in TNF-α and IL-6 levels in LPS-activated cells, when compared to controls LPS-activated cells (Fig 3).

### Gene expression

The data on the relative transcription of genes regulated by BRP in LPS-activated macrophages compared with control LPS activated cells (treated with vehicle -DMSO) are shown in Table 1. The transcription of *Cd80*, *Cd86*, *Naip1* was up-regulated by BRP in LPS activated macrophages, whereas transcription of *Ccnd1*, *Cd14*, *Eif2ak2*, *Elk1*, *Flt3l*, *Fos*, *Gdf1*, *Il10*, *Il1b*, *Il1f9*, *Il1rn*, *Map2k1*, *Mapk14*, *Mcr*, *Mtcp1*, *Naip1*, *Nfkb1*, *Pak1*, *Pdk1*, *Pik3ca*, *Pik3cg*, *Pik3r2*, *Prkca*, *Rps6ka1*, *Srf*, *Tirap*, *Tlr4* and *Tnfsf12* were down-regulated, among 360 studied genes involved in the inflammatory process.

**Table 1:** List of genes of RAW 264.7 cells activated with LPS (500 ng/mL) which were regulated by the treatment with 50 μg BRP/mL. **Fold changes were calculated in relation to LPS activated cells with and treated with DMSO (control). E**xperiments performed in triplicate.

Gene	Fold-change	*P* value	Function
*Ccnd1*	-3.1	0.019304	PI3K/AKT pathway [27]
*Cd14*	-2.1	0.00953	TLR response [28]
*Cd80*	4.7	0.03	Macrophage polarization marker [8, 29]
*Cd86*	4.3	0.02	Macrophage polarization marker[8, 29]
*Eif2ak2*	-2.3	0.006488	NF-κB pathway [30]
*Elk1*	-7.3	0.000049	TLR response [31] and cancer development [32]
*Flt3l*	-4.1	0.00104	PI3K/AKT pathway [33]
*Fos*	-5.9	0.00083	Cancer development [34]
*Gdf1*	-2.7	7E-06	TGF-beta super-family [35]
*Il10*	-6.0	0.03872	Anti-inflammatory cytokine [36].
*Il1b*	-6.2	0.050638	Inflammatory cytokine [37, 38]
*Il1f9*	-27.5	0.03639	Inflammatory cytokine [39]
*Il1rn*	-4.2	0.00048	IL-1 pathway [40]
*Map2k1*	-3.3	0.032279	MAPK pathway [41]
*Mapk14*	-4.1	0.014773	MAPK pathway [42]
*Mcr*	-2.5	0.04	Macrophage polarization marker[8, 29]
*Mtcp1*	-7.9	0.000981	Increase activation of AKT1 and AKT2 [43]
*Naip1*	6.3	0.032882	Anti-apoptotic [44]
*Nfkb1*	-5.8	0.05567	NF-κB pathway [45]
*Pak1*	-17.3	0.032134	MAPK pathway [46]
*Pdk1*	-5.6	0.031172	NO pathway [47]
*Pik3ca*	-2.1	0.024495	PI3K/AKT pathway[48] and involved in cancer [49]
*Pik3cg*	-2.7	0.008274	PI3K/AKT pathway and TLR response [50]
*Pik3r2*	-2.6	0.020004	PI3K/AKT pathway [48]
*Prkca*	-4.7	0.000282	MAPK pathway [51]
*Rps6ka1*	-2.2	0.017273	MAPK pathway [52]
*Srf*	-4.8	0.038967	MAPK pathway [53]
*Tirap*	-47.9	0.022142	TLR response [54]
*Tlr4*	-3.6	0.018578	TLR response [55]
*Tnfsf12*	-2.6	0.00383	TNF-α cascade [56]

### Signaling pathways analysis and TIRAP expression

**Fig 4:**
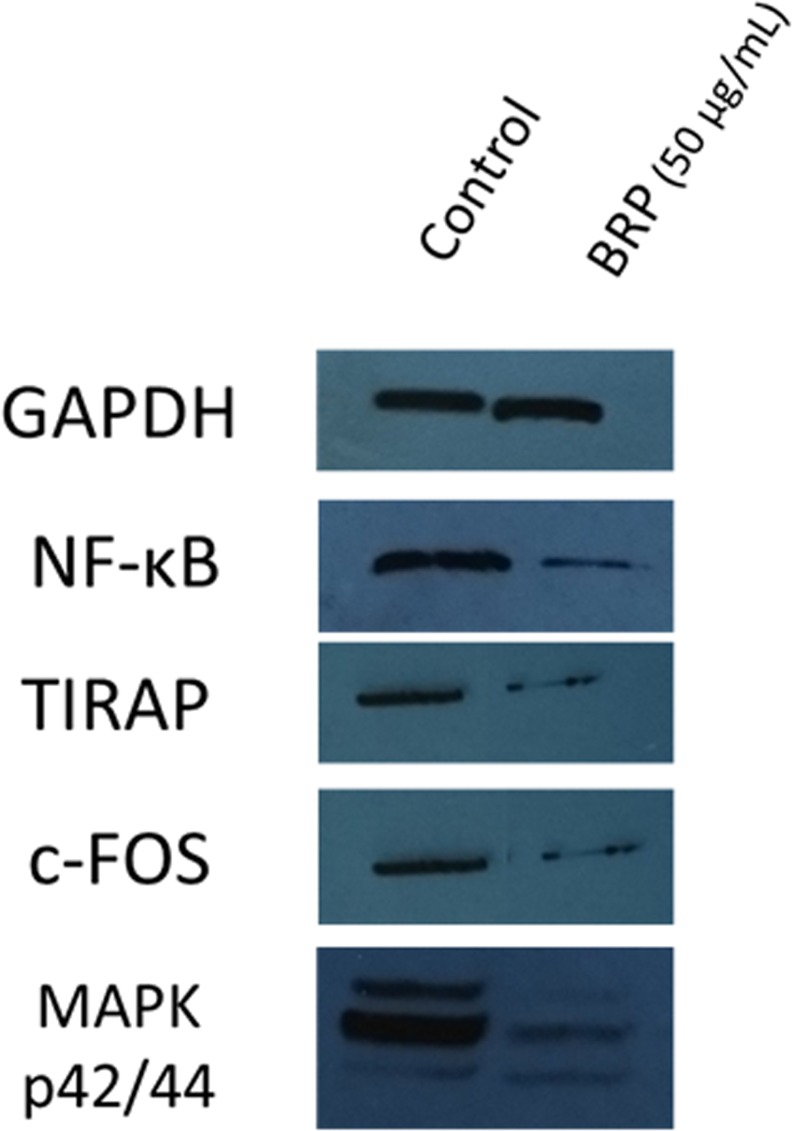
Western blot image showing decreased levels of phosphorylated NF-κB, p65 sub-unit, c-FOS and MAPK p42/44, after treatment of LPS-activated RAW 264.7 macrophages with BRP. Decreased levels of the adaptor TIRAP is also shown. GAPDH was used as control.

Western blot assays revealed that BRP (50 μg / ml) treatment decreased the relative levels of the following phosphorylated proteins NF-κB, C-FOS and MAPK p42/44 when normalized to GAPDH levels (Fig 4) indicating that BRP inhibits several signaling pathways. Furthermore, TIRAP levels were also reduced by BRP.

## Discussion

Inflammation must be tightly controlled in order to respond to harmful threats without causing tissue damage [29]. Monocytes derived macrophages can be recruited to target tissues during inflammation and pathogen challenge. These cells can display remarkable phenotypic heterogeneity playing different roles depending on the environment [57].

In response to an infectious challenge, bacterial components such as LPS induce monocytes differentiation into classically activated macrophages or M1, in order to kill pathogens via phagocytosis, production of reactive oxygen species, nitric oxide enzymes and inflammatory cytokines[29]. Our data indicated that BRP does not interfere in non-activated monocytes, with no effect on cell viability neither on NO production (Fig 2A). BRP treated LPS activated macrophages were polarized to M1 phenotype, and this polarization was even more significant, since transcription of *cd80* and *cd86* was up-regulated, and of *mrc1* down-regulated, and the production of TNF-α and IL-6 was slightly increased in LPS-macrophages treated with BRP than in those only activated by LPS

However, when compared to LPS-treated control macrophages, BRP led to reduced production of pro-inflammatory factors such as NO, IL-12, IL-1β, GM-CSF, and several genes associated with inflammation were down-regulated, evidencing the role of BRP in modulating the macrophages response to LPS. Granulocyte-macrophage colony-stimulating factor (GM-CSF) is involved in the development, differentiation, and proliferation of macrophages during the inflammatory state, leading to the M1-like inflammatory phenotype[29] and its reduction by BRP may be associated with the altered phenotype of macrophages.

The transcription analyses of BRP treated LPS-activated macrophages showed the inhibition of at least four pro-inflammatory pathways in relation to control LPS-activated macrophages. BRP inhibited IL-1β pathway due to down-regulation of *Il1b* (encoding for IL-1β) and *Il1f9* (encoding IL-36γ)[39], which was evidenced by reduced IL-1β levels in the cell supernatant.

IL-1 inhibition is noteworthy for its anti-inflammatory properties [1] leading to inhibition of a cascade that activates nuclear factor kappa B (NF-κB) pathway [37], nitric oxide synthase (iNOS)[38], and production of pro-inflammatory cytokines. IL-36γ is a member of the IL-1 family involved in IL-1 independent inflammatory response, but its role in homeostasis or pathogenesis is still under discussion [58]. IL-36γ is expressed by THP-1 macrophages after LPS stimulus, and activates NFκB and Mitogen-Activated Protein Kinase (MAPK) pathways [58]. On the other hand, possibly in response to IL-1β pathway inhibition by BRP, *il1rn*, encoding the antagonist receptor of IL-1 was also down- regulated (-4.2 fold changes) [40], contradicting BRP anti-inflammatory properties. Thus, the effect of BRP in IL-1 and IL-36γ pathways may have mediated the inhibition of downstream pathways including NFκB and MAPK inhibition in LPS-activated macrophages and consequently the production of NO, and pro-inflammatory cytokines.

The decrease in NF-κB signaling pathway (Fig 4) promoted by BRP resulted in reduced expression of *Eif2ak*, *Nfkb1*, *Il1b*, *Il1f9* and *Tnfsf12* [30, 37, 39, 45, 56]. Furthermore, reduced activation of MAPK pathway was indicated not only by reduced phosphorylation of MAPK42/44 but also by the down-regulation of *Map2k1*, *Mapk14*, *Pak1*, *Prkca*, *Rps6ka1*, *Srf* [41, 42, 46, 51–53]. The negative regulation of *Mapk14* is in accordance with the reduction of IL-12 levels, since MAPK 14 induces the production of IL-12[59]. Moreover, decreased activation of PI3K/AKT pathway by BRP was achieved, since *Ccnd1*, *Mtcp1*, *Pik3ca*, *Pik3cg*, *Pik3r2*, *Flt3L* were also down-regulated in BRP treated LPS-activated macrophages [27, 33, 43, 48].

BRP treated LPS-activated macrophages demonstrated low production of NO (Fig 2C), which is consistent with the inhibition of the NO pathway, inhibition of NF-κB, and decrease in IL-1 production in the BRP treated LPS-activated macrophages [38]. Furthermore the down-regulation of *Pdk1* may also contribute with this reduction, since PDK1 inhibition leads to inhibition of eNOS (constitutive nitric oxide synthase)[47].

The anti-inflammatory mechanism of BRP was also shown by the down-regulation of transcription of other genes correlated with inflammation, which are usually up-regulated in inflammatory diseases. The mRNA levels of *Tnfsf12*, which encodes Tweak (TNF-like weak inducer of apoptosis), were also reduced in BRP treated LPS-activated macrophages. After binding to its receptor Fn14, Tweak signals through a variety of downstream signaling cascades, including the NF-κB, MAPK, and AKT pathways [60]. Furthermore, a remarkable Tweak expression can be observed in monocytes upon stimulation with interferon (IFN)-γ but not with lipopolysaccharide [61]. Thus, the diminished expression of *Tnfsf12* promoted by BRP may be the result of inhibition of IFNγ production.

**Fig 5:**
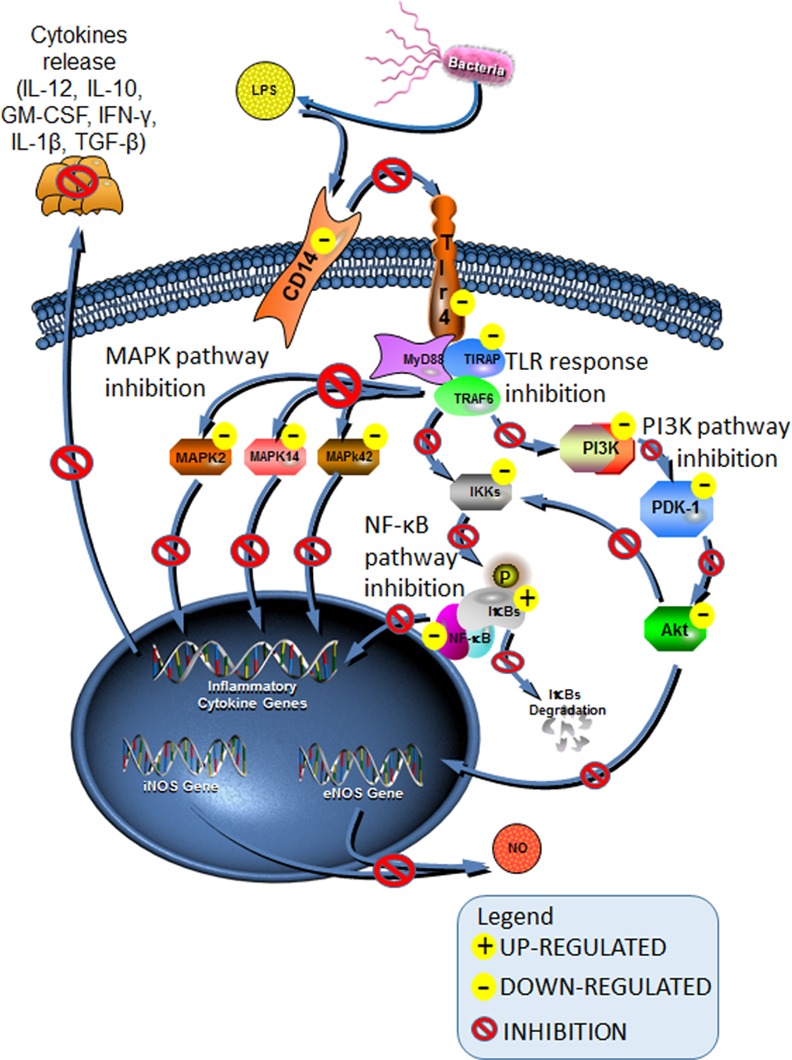
Brazilian red propolis anti-inflammatory molecular mechanisms in LPS activated macrophages. “-” means that transcription of genes and/or pathway activation were diminished by BRP. “+” that transcription of genes and/or pathway activation were increased by BRP. LPS-activated macrophages are polarized in M1, but BRP treatment promoted an altered M1 phenotype. BRP led to inhibition of genes related to Toll-like receptor (*Cd14*, *Elk1*, *Pik3cg*, *Tirap* and *Tlr4*). The resulting attenuation of TLR-mediated signaling led to the inhibition of NFκB, Mitogen-Activated Protein Kinase (MAPK) and PI3K/AKT pathways. Thus BRP decreased the production of cytokines and nitric oxide, involved in the inflammatory process. Adapted from Qiagen’s website (https://www.qiagen.com/br/shop/genes-and-pathways/pathway-central/?q=).

BRP strongly down-regulated the expression of genes related to Toll-like receptor (TLR) response (*Cd14*, *Elk1*, *Pik3cg*, *Tirap* and *Tlr4*). The attenuation of TLR-mediated signaling pathways in LPS activated macrophages treated with BRP was confirmed by the reduction in the levels of toll-interleukin 1 receptor (TIR) domain containing adaptor protein (TIRAP) [54]. TIRAP/Mal is critically involved in the MyD88-dependent pathway, via TLR4 and TLR2 [62]. In addition, TIRAP also acts via TLR1 and TLR 6 activation [63]. Previous studies revealed that TIRAP/Mal knockout macrophages showed impaired inflammatory cytokine production and delayed activation of JNK and NF-κB in response to the TLR4 ligand. It is relevant to note that resveratrol, known for its cardioprotective, anti-cancer, anti-oxidant, anti-inflammatory, anti-diabetes, anti-obesity, anti-Alzheimer and anti-Parkinson effects, also suppresses the expression of TIRAP[64], and a similar effect may be expected from BRP. Therefore, our data demonstrated that propolis may decrease the macrophages response to LPS and in consequence, may control the inflammation and modulate its harmful effects to the organism, as summarized in Fig 5.

Surprisingly, IL-10 was strongly repressed by BRP at the mRNA and protein levels in LPS-activated macrophages. IL-10 couteracts the proinflammatory cytokines induced earlier by LPS activated macrophages, by triggering secondary signaling pathways, which modulate the expression of direct LPS target genes, although the anti-inflammatory properties of IL-10 are still controversial [65]. Thus, IL-10 down-regulation promoted by BRP may have led to the slightly increase in TNF-α levels seem in the cell supernatants [36].

The anti-inflammatory mechanisms induced by BRP, that we have shown, could be due to the complex chemical profile of this product [12, 15] which includes isoflavones, known for their anti-inflammatory, antimicrobial and antioxidant effects [14, 17, 66–68]. At least 20 different compounds could be identified in BRP (Fig 1), of which vestitol and neovestitol were the major components. In this way, future studies should isolate BRP compounds, as performed by Inui et al. (2014) [69] and Bueno-Silva et al. (2013a,b) [14, 17], in order to determinate which fraction or compound(s) is responsible for the BRP modulatory effect. This chemical diversity confirmed the value of BRP in drug discovery, turning BRP into an important food-source of new compounds with therapeutic properties as a nutraceutical that could be used by the food and pharmaceutical industries.

In addition, our data on gene expression revealed new possible biological uses of red propolis. BRP negatively regulated the expression of numerous genes involved in the development of several types of cancer such as: *fos* [34], *elk1* [32], *Pik3ca* [49], *Prkca* [51]. On the other hand, the cells were protected from apoptosis by up regulation of *naip1*, encoding the anti-apoptotic protein Naip1, which inhibits caspases 3, 7 and 9 [44].

The classification of macrophages polarization as M1/M2 is limited, and as shown here macrophages can adopt multiple phenotypes according to the stimulus in the environment. The present data indicated that BRP alters the signaling promoted by LPS in monocyte-derived macrophages, inducing a lower production of proinflammatory mediators, such as IL-1 and IL-12 but not of TNF-α, by interfering with the TLR response and leading to inhibition of NF-κB, MAPK and PI3K signaling pathways. The effect of BRP on macrophages activation suggests its potential as food-source of new compounds with pharmacological properties and its use in the control of pathological inflammation.

## References

[1] Dinarello CA, Simon A, van der Meer JW. Treating inflammation by blocking interleukin-1 in a broad spectrum of diseases. Nature reviews Drug discovery. 2012;11(8):633–52. 10.1038/nrd3800 22850787 PMC3644509

[2] Matsumoto H, Haga K, Ohno I, Hiraoka K, Kimura T, Hermann K, et al Mucosal gene therapy using a pseudotyped lentivirus vector encoding murine interleukin-10 (mIL-10) suppresses the development and relapse of experimental murine colitis. BMC gastroenterology. 2014;14:68 10.1186/1471-230X-14-68 24712338 PMC3991919

[3] Smolen JS, Weinblatt ME, Sheng S, Zhuang Y, Hsu B. Sirukumab, a human anti-interleukin-6 monoclonal antibody: a randomised, 2-part (proof-of-concept and dose-finding), phase II study in patients with active rheumatoid arthritis despite methotrexate therapy. Annals of the rheumatic diseases. 2014;73(9):1616–25. 10.1136/annrheumdis-2013-205137 24699939 PMC4145446

[4] Guilliams M, Ginhoux F, Jakubzick C, Naik SH, Onai N, Schraml BU, et al Dendritic cells, monocytes and macrophages: a unified nomenclature based on ontogeny. Nature reviews Immunology. 2014;14(8):571–8. 10.1038/nri3712 . 25033907 PMC4638219

[5] Epelman S, Lavine KJ, Randolph GJ. Origin and functions of tissue macrophages. Immunity. 2014;41(1):21–35. 10.1016/j.immuni.2014.06.013 25035951 PMC4470379

[6] Murray PJ, Allen JE, Biswas SK, Fisher EA, Gilroy DW, Goerdt S, et al Macrophage activation and polarization: nomenclature and experimental guidelines. Immunity. 2014;41(1):14–20. 10.1016/j.immuni.2014.06.008 25035950 PMC4123412

[7] Labonte AC, Tosello-Trampont AC, Hahn YS. The role of macrophage polarization in infectious and inflammatory diseases. Molecules and cells. 2014;37(4):275–85. 10.14348/molcells.2014.2374 24625576 PMC4012075

[8] Martinez FO, Sica A, Mantovani A, Locati M. Macrophage activation and polarization. Frontiers in bioscience: a journal and virtual library. 2008;13:453–61. . 17981560 10.2741/2692

[9] Mackova A, Mucaji P, Widowitz U, Bauer R. In vitro anti-inflammatory activity of Ligustrum vulgare extracts and their analytical characterization. Natural product communications. 2013;8(11):1509–12. . 24427928

[10] Saravanan S, Islam VI, Babu NP, Pandikumar P, Thirugnanasambantham K, Chellappandian M, et al Swertiamarin attenuates inflammation mediators via modulating NF-kappaB/I kappaB and JAK2/STAT3 transcription factors in adjuvant induced arthritis. European journal of pharmaceutical sciences: official journal of the European Federation for Pharmaceutical Sciences. 2014;56:70–86. 10.1016/j.ejps.2014.02.005 . 24582615

[11] Cragg GM, Newman DJ. Natural products: a continuing source of novel drug leads. Biochimica et biophysica acta. 2013;1830(6):3670–95. 10.1016/j.bbagen.2013.02.008 23428572 PMC3672862

[12] Silva BB, Rosalen PL, Cury JA, Ikegaki M, Souza VC, Esteves A, et al Chemical composition and botanical origin of red propolis, a new type of brazilian propolis. Evidence-based complementary and alternative medicine. 2008;5(3):313–6. 10.1093/ecam/nem059 18830449 PMC2529384

[13] da Cunha MG, Franchin M, Galvao LC, Bueno-Silva B, Ikegaki M, de Alencar SM, et al Apolar bioactive fraction of Melipona scutellaris geopropolis on *Streptococcus mutans* biofilm. Evidence-based complementary and alternative medicine. 2013;2013:256287 10.1155/2013/256287 23843868 PMC3697201

[14] Bueno-Silva B, Koo H, Falsetta ML, Alencar SM, Ikegaki M, Rosalen PL. Effect of neovestitol-vestitol containing Brazilian red propolis on accumulation of biofilm in vitro and development of dental caries in vivo. Biofouling. 2013;29(10):1233–42. 10.1080/08927014.2013.834050 24099330 PMC3855307

[15] Oldoni TLC, Cabral ISR, d'Arce MABR, Rosalen PL, Ikegaki M, Nascimento AM, et al Isolation and analysis of bioactive isoflavonoids and chalcone from a new type of Brazilian propolis. Sep Purif Technol. 2011;77(2):208–13. 10.1016/j.seppur.2010.12.007 .

[16] Ishihara M, Naoi K, Hashita M, Itoh Y, Suzui M. Growth inhibitory activity of ethanol extracts of Chinese and Brazilian propolis in four human colon carcinoma cell lines. Oncology reports. 2009;22(2):349–54. 10.3892/or_00000444 . 19578776

[17] Bueno-Silva B, Alencar SM, Koo H, Ikegaki M, Silva GVJ, Napimoga MH, et al Anti-inflammatory and antimicrobial evaluation of neovestitol and vestitol isolated from Brazilian red propolis. J Agr Food Chem. 2013;61(19):4546–50. 10.1021/Jf305468f . 23607483

[18] Franchin M, da Cunha MG, Denny C, Napimoga MH, Cunha TM, Koo H, et al Geopropolis from Melipona scutellaris decreases the mechanical inflammatory hypernociception by inhibiting the production of IL-1beta and TNF-alpha. J Ethnopharmacol. 2012;143(2):709–15. 10.1016/j.jep.2012.07.040 . 22885134

[19] Park YK, Alencar SM, Aguiar CL. Botanical origin and chemical composition of Brazilian propolis. J Agr Food Chem. 2002;50(9):2502–6. 10.1021/jf011432b . 11958612

[20] Lopez BG, Schmidt EM, Eberlin MN, Sawaya AC. Phytochemical markers of different types of red propolis. Food Chem. 2014;146:174–80. 10.1016/j.foodchem.2013.09.063 . 24176329

[21] Paulino N, Abreu SR, Uto Y, Koyama D, Nagasawa H, Hori H, et al Anti-inflammatory effects of a bioavailable compound, Artepillin C, in Brazilian propolis. Eur J Pharmacol. 2008;587(1–3):296–301. 10.1016/j.ejphar.2008.02.067 . 18474366

[22] Paulino N, Teixeira C, Martins R, Scremin A, Dirsch VM, Vollmar AM, et al Evaluation of the analgesic and anti-inflammatory effects of a Brazilian green propolis. Planta Med. 2006;72(10):899–906. 10.1055/s-2006-947185 . 16902858

[23] Lima LD, Andrade SP, Campos PP, Barcelos LS, Soriani FM, Moura SA, et al Brazilian green propolis modulates inflammation, angiogenesis and fibrogenesis in intraperitoneal implant in mice. BMC Complement Altern Med. 2014;14:177 10.1186/1472-6882-14-177 24886376 PMC4061536

[24] Machado JL, Assuncao AK, da Silva MC, Dos Reis AS, Costa GC, Arruda Dde S, et al Brazilian green propolis: anti-inflammatory property by an immunomodulatory activity. Evidence-based complementary and alternative medicine: eCAM. 2012;2012:157652 10.1155/2012/157652 23320022 PMC3541042

[25] Miranda MM, Panis C, Cataneo AH, da Silva SS, Kawakami NY, Lopes LG, et al Nitric oxide and Brazilian propolis combined accelerates tissue repair by modulating cell migration, cytokine production and collagen deposition in experimental leishmaniasis. Plos One. 2015;10(5):e0125101 10.1371/journal.pone.0125101 25973801 PMC4431861

[26] Ando-Suguimoto ES, da Silva MP, Kawamoto D, Chen C, DiRienzo JM, Mayer MP. The cytolethal distending toxin of Aggregatibacter actinomycetemcomitans inhibits macrophage phagocytosis and subverts cytokine production. Cytokine. 2014;66(1):46–53. 10.1016/j.cyto.2013.12.014 . 24548424

[27] Qiu C, Xie Q, Zhang D, Chen Q, Hu J, Xu L. GM-CSF induces cyclin D1 expression and proliferation of endothelial progenitor cells via PI3K and MAPK signaling. Cellular physiology and biochemistry: international journal of experimental cellular physiology, biochemistry, and pharmacology. 2014;33(3):784–95. 10.1159/000358652 . 24662605

[28] Kelley SL, Lukk T, Nair SK, Tapping RI. The crystal structure of human soluble CD14 reveals a bent solenoid with a hydrophobic amino-terminal pocket. J Immunol. 2013;190(3):1304–11. 10.4049/jimmunol.1202446 23264655 PMC3552104

[29] Italiani P, Boraschi D. From Monocytes to M1/M2 Macrophages: Phenotypical vs. Functional Differentiation. Frontiers in immunology. 2014;5:514 10.3389/fimmu.2014.00514 25368618 PMC4201108

[30] Garcia MA, Gil J, Ventoso I, Guerra S, Domingo E, Rivas C, et al Impact of protein kinase PKR in cell biology: from antiviral to antiproliferative action. Microbiology and molecular biology reviews: MMBR. 2006;70(4):1032–60. 10.1128/MMBR.00027-06 17158706 PMC1698511

[31] Hodgkinson CP, Laxton RC, Patel K, Ye S. Advanced glycation end-product of low density lipoprotein activates the toll-like 4 receptor pathway implications for diabetic atherosclerosis. Arteriosclerosis, thrombosis, and vascular biology. 2008;28(12):2275–81. 10.1161/ATVBAHA.108.175992 . 18818414

[32] Besnard A, Galan-Rodriguez B, Vanhoutte P, Caboche J. Elk-1 a transcription factor with multiple facets in the brain. Frontiers in neuroscience. 2011;5:35 10.3389/fnins.2011.00035 21441990 PMC3060702

[33] Sathaliyawala T, O'Gorman WE, Greter M, Bogunovic M, Konjufca V, Hou ZE, et al Mammalian target of rapamycin controls dendritic cell development downstream of Flt3 ligand signaling. Immunity. 2010;33(4):597–606. 10.1016/j.immuni.2010.09.012 20933441 PMC2966531

[34] Fialka I, Schwarz H, Reichmann E, Oft M, Busslinger M, Beug H. The estrogen-dependent c-JunER protein causes a reversible loss of mammary epithelial cell polarity involving a destabilization of adherens junctions. The Journal of cell biology. 1996;132(6):1115–32. 8601589 10.1083/jcb.132.6.1115 PMC2120757

[35] Rankin CT, Bunton T, Lawler AM, Lee SJ. Regulation of left-right patterning in mice by growth/differentiation factor-1. Nature genetics. 2000;24(3):262–5. 10.1038/73472 . 10700179

[36] Williams LM, Ricchetti G, Sarma U, Smallie T, Foxwell BM. Interleukin-10 suppression of myeloid cell activation—a continuing puzzle. Immunology. 2004;113(3):281–92. 10.1111/j.1365-2567.2004.01988.x 15500614 PMC1782589

[37] Janssens S, Burns K, Tschopp J, Beyaert R. Regulation of interleukin-1- and lipopolysaccharide-induced NF-kappaB activation by alternative splicing of MyD88. Current biology: CB. 2002;12(6):467–71. . 11909531 10.1016/s0960-9822(02)00712-1

[38] Hewett SJ, Corbett JA, McDaniel ML, Choi DW. Interferon-gamma and interleukin-1 beta induce nitric oxide formation from primary mouse astrocytes. Neuroscience letters. 1993;164(1–2):229–32. . 7512249 10.1016/0304-3940(93)90898-u

[39] Foster AM, Baliwag J, Chen CS, Guzman AM, Stoll SW, Gudjonsson JE, et al IL-36 promotes myeloid cell infiltration, activation, and inflammatory activity in skin. J Immunol. 2014;192(12):6053–61. 10.4049/jimmunol.1301481 24829417 PMC4048788

[40] Butcher C, Steinkasserer A, Tejura S, Lennard AC. Comparison of two promoters controlling expression of secreted or intracellular IL-1 receptor antagonist. J Immunol. 1994;153(2):701–11. . 8021506

[41] Burgermeister E, Chuderland D, Hanoch T, Meyer M, Liscovitch M, Seger R. Interaction with MEK causes nuclear export and downregulation of peroxisome proliferator-activated receptor gamma. Molecular and cellular biology. 2007;27(3):803–17. 10.1128/MCB.00601-06 17101779 PMC1800691

[42] Chan ED, Morris KR, Belisle JT, Hill P, Remigio LK, Brennan PJ, et al Induction of inducible nitric oxide synthase-NO* by lipoarabinomannan of Mycobacterium tuberculosis is mediated by MEK1-ERK, MKK7-JNK, and NF-kappaB signaling pathways. Infection and immunity. 2001;69(4):2001–10. 10.1128/IAI.69.4.2001-2010.2001 11254551 PMC98123

[43] Laine J, Kunstle G, Obata T, Noguchi M. Differential regulation of Akt kinase isoforms by the members of the TCL1 oncogene family. The Journal of biological chemistry. 2002;277(5):3743–51. 10.1074/jbc.M107069200 . 11707444

[44] Davoodi J, Ghahremani MH, Es-Haghi A, Mohammad-Gholi A, Mackenzie A. Neuronal apoptosis inhibitory protein, NAIP, is an inhibitor of procaspase-9. The international journal of biochemistry & cell biology. 2010;42(6):958–64. 10.1016/j.biocel.2010.02.008 . 20171302

[45] Hayden MS, West AP, Ghosh S. NF-kappaB and the immune response. Oncogene. 2006;25(51):6758–80. 10.1038/sj.onc.1209943 . 17072327

[46] Tang Y, Marwaha S, Rutkowski JL, Tennekoon GI, Phillips PC, Field J. A role for Pak protein kinases in Schwann cell transformation. Proceedings of the National Academy of Sciences of the United States of America. 1998;95(9):5139–44. 9560242 10.1073/pnas.95.9.5139 PMC20227

[47] Wei Q, Xia Y. Roles of 3-phosphoinositide-dependent kinase 1 in the regulation of endothelial nitric-oxide synthase phosphorylation and function by heat shock protein 90. The Journal of biological chemistry. 2005;280(18):18081–6. 10.1074/jbc.M413607200 . 15737995

[48] Barberis L, Hirsch E. Targeting phosphoinositide 3-kinase gamma to fight inflammation and more. Thrombosis and haemostasis. 2008;99(2):279–85. 10.1160/TH07-10-0632 . 18278175

[49] Stein RC. Prospects for phosphoinositide 3-kinase inhibition as a cancer treatment. Endocrine-related cancer. 2001;8(3):237–48. . 11566615 10.1677/erc.0.0080237

[50] Kim DI, Kim SR, Kim HJ, Lee SJ, Lee HB, Park SJ, et al PI3K-gamma inhibition ameliorates acute lung injury through regulation of IkappaBalpha/NF-kappaB pathway and innate immune responses. Journal of clinical immunology. 2012;32(2):340–51. 10.1007/s10875-011-9628-1 . 22198681

[51] St-Denis A, Chano F, Tremblay P, St-Pierre Y, Descoteaux A. Protein kinase C-alpha modulates lipopolysaccharide-induced functions in a murine macrophage cell line. The Journal of biological chemistry. 1998;273(49):32787–92. . 9830023 10.1074/jbc.273.49.32787

[52] Dalby KN, Morrice N, Caudwell FB, Avruch J, Cohen P. Identification of regulatory phosphorylation sites in mitogen-activated protein kinase (MAPK)-activated protein kinase-1a/p90rsk that are inducible by MAPK. The Journal of biological chemistry. 1998;273(3):1496–505. . 9430688 10.1074/jbc.273.3.1496

[53] Wu SQ, Minami T, Donovan DJ, Aird WC. The proximal serum response element in the Egr-1 promoter mediates response to thrombin in primary human endothelial cells. Blood. 2002;100(13):4454–61. 10.1182/blood-2002-02-0415 . 12393577

[54] Bonham KS, Orzalli MH, Hayashi K, Wolf AI, Glanemann C, Weninger W, et al A promiscuous lipid-binding protein diversifies the subcellular sites of toll-like receptor signal transduction. Cell. 2014;156(4):705–16. 10.1016/j.cell.2014.01.019 24529375 PMC3951743

[55] Kawai T, Akira S. Signaling to NF-kappaB by Toll-like receptors. Trends in molecular medicine. 2007;13(11):460–9. 10.1016/j.molmed.2007.09.002 . 18029230

[56] Wiley SR, Winkles JA. TWEAK, a member of the TNF superfamily, is a multifunctional cytokine that binds the TweakR/Fn14 receptor. Cytokine & growth factor reviews. 2003;14(3–4):241–9. . 12787562 10.1016/s1359-6101(03)00019-4

[57] Okabe Y, Medzhitov R. Tissue-specific signals control reversible program of localization and functional polarization of macrophages. Cell. 2014;157(4):832–44. 10.1016/j.cell.2014.04.016 24792964 PMC4137874

[58] Gresnigt MS, van de Veerdonk FL. Biology of IL-36 cytokines and their role in disease. Seminars in immunology. 2013;25(6):458–65. 10.1016/j.smim.2013.11.003 . 24355486

[59] Lee SM, Kim EJ, Suk K, Lee WH. BAFF and APRIL induce inflammatory activation of THP-1 cells through interaction with their conventional receptors and activation of MAPK and NF-kappaB. Inflammation research: official journal of the European Histamine Research Society [et al]. 2011;60(9):807–15. 10.1007/s00011-011-0336-3 . 21505913

[60] Burkly LC, Kawashima R, Kawamura YI, Oshio T, Son A, Yamazaki M, et al TWEAK/Fn14 Pathway in Colitis: Links to Interleukin-13-induced intestinal epithelial cell injury and promotion of chronic colitis. Cytokine. 2011;56(1):109–. 10.1016/j.cyto.2011.07.425 .

[61] Nakayama M, Kayagaki N, Yamaguchi N, Okumura K, Yagita H. Involvement of TWEAK in interferon gamma-stimulated monocyte cytotoxicity. The Journal of experimental medicine. 2000;192(9):1373–80. 11067885 10.1084/jem.192.9.1373 PMC2193363

[62] Takeda K, Akira S. TLR signaling pathways. Seminars in immunology. 2004;16(1):3–9. . 14751757 10.1016/j.smim.2003.10.003

[63] Bernard NJ, O'Neill LA. Mal, more than a bridge to MyD88. IUBMB life. 2013;65(9):777–86. 10.1002/iub.1201 . 23983209

[64] Kim S, Jin Y, Choi Y, Park T. Resveratrol exerts anti-obesity effects via mechanisms involving down-regulation of adipogenic and inflammatory processes in mice. Biochemical pharmacology. 2011;81(11):1343–51. 10.1016/j.bcp.2011.03.012 . 21439945

[65] Rossol M, Heine H, Meusch U, Quandt D, Klein C, Sweet MJ, et al LPS-induced cytokine production in human monocytes and macrophages. Critical reviews in immunology. 2011;31(5):379–446. . 22142165 10.1615/critrevimmunol.v31.i5.20

[66] Bandara M, Arun SJ, Allanson M, Widyarini S, Chai Z, Reeve VE. Topical isoflavonoids reduce experimental cutaneous inflammation in mice. Immunol Cell Biol. 2010;88(7):727–33. 10.1038/icb.2010.26 . 20212509

[67] Gupta C, Prakash D. Phytonutrients as therapeutic agents. Journal of complementary & integrative medicine. 2014;11(3):151–69. 10.1515/jcim-2013-0021 . 25051278

[68] Lepri SR, Zanelatto LC, da Silva PB, Sartori D, Ribeiro LR, Mantovani MS. Effects of genistein and daidzein on cell proliferation kinetics in HT29 colon cancer cells: the expression of CTNNBIP1 (beta-catenin), APC (adenomatous polyposis coli) and BIRC5 (survivin). Human cell. 2014;27(2):78–84. 10.1007/s13577-012-0051-6 . 24390805

[69] Inui S, Hatano A, Yoshino M, Hosoya T, Shimamura Y, Masuda S, et al Identification of the phenolic compounds contributing to antibacterial activity in ethanol extracts of Brazilian red propolis. Natural product research. 2014;28(16):1293–6. 10.1080/14786419.2014.898146 . 24666260

